# Community-acquired bacterial meningitis in Southern Sweden 2013–2023: a population-based study of incidence, aetiology and diagnostic yield

**DOI:** 10.1007/s10096-025-05247-w

**Published:** 2025-10-01

**Authors:** Tobias West, Robin Carlander, Torgny Sunnerhagen, Gustav Torisson, Oskar Ljungquist

**Affiliations:** 1 Division of Infection Medicine, Department of Clinical Sciences, Faculty of Medicine, Lund University, Lund, Sweden; 2 Department of Internal Medicine, Helsingborg hospital, Helsingborg, Sweden; 3 Department of Clinical Microbiology, Infection Control and Prevention, Skåne University Hospital, Lund, Sweden; 4 Clinical Infection Medicine, Department of Translational Medicine, Lund University, Malmö, Sweden; 5 Department of Infectious Diseases, Skåne University Hospital, Malmö, Sweden; 6 Department of Infectious Diseases, Helsingborg hospital, Helsingborg, Sweden

**Keywords:** Bacterial meningitis, Incidence, Aetiology, Serotypes, Vaccine-preventable, Temporal trend

## Abstract

**Purpose:**

Despite advances in medical care, bacterial meningitis still poses a considerable health issue from a global perspective. An ageing population and increasing development and use of vaccines are likely to affect the incidence and aetiology. The aim of this study was to describe the incidence and aetiology of community-acquired bacterial meningitis (CABM) in our setting, as well as the serotypes of *Streptococcus pneumoniae* and *Neisseria meningitidis* causing CABM, in relation to available vaccines.

**Method:**

Patients of all ages with CABM in southern Sweden 2013**–**2023 were included. Patients were identified through records of cerebrospinal fluid tests from the Department of Clinical Microbiology, and through International Classification of Diseases 10 codes for bacterial meningitis. Age-standardised incidence rates were calculated based on the European Standard Population 2013.

**Results:**

During the study period, 244 episodes of CABM in 238 individuals were identified. Definitive aetiology could be established in 93% of episodes. Mean incidence rate was 1.63 per 100,000 person-years, with a trend of declining incidence during the study period. *Streptococcus pneumoniae* was the most common pathogen, accounting for 46.7% of episodes. 63.6% of the episodes of pneumococcal meningitis were caused by serotypes included in current vaccines.

**Conclusion:**

Pneumococcal meningitis is the primary driver of incidence and trends of all-cause community-acquired bacterial meningitis in southern Sweden. Further studies are warranted to investigate how vaccination could influence both disease incidence and shifts in serotype distribution, while also identifying optimal patient populations for targeted interventions.

**Supplementary Information:**

The online version contains supplementary material available at 10.1007/s10096-025-05247-w.

## Introduction

Despite advances in medical care and vaccination, bacterial meningitis still presents a substantial global health issue with considerable mortality and morbidity. In 2019, meningitis was estimated to cause 2.51 million cases and 236,000 deaths globally, of which a majority was caused by bacterial pathogens [1]. A recent meta-analysis [2] reported a case fatality rate of 15% for bacterial meningitis and 20% of survivors are affected by long-term sequelae [3]. This encouraged the World Health Organization to initiate a global project with the goal to reduce cases of vaccine-preventable meningitis by 50% and deaths by 70% by year 2030 [4].

Incidence rates and aetiology vary considerably throughout the world, depending on access to vaccines, age distribution in the population, and regional epidemics [5]. More recent studies from Europe and the US report incidence rates < 1.0 per 100,000 person-years [6–8]. The incidence rates are significantly higher in low-income countries in Africa and Asia, in some regions exceeding 100 per 100,000 person-years [1, 9]. The true incidence in many low-income countries is uncertain, however, due to limited access to diagnostic methods, less developed surveillance programs, health care and vaccine accessibility, and differences in health-seeking behaviour [4].

From a global perspective, *Streptococcus pneumoniae*, *Neisseria meningitidis*, and *Haemophilus influenzae* account for most cases of bacterial meningitis [1]. However, since the development of vaccines against certain subtypes, the relative burden of disease has shifted in countries where vaccines are in use. To some extent, vaccine-preventable serotypes are replaced by other serotypes [10]. Among neonates, *Streptococcus agalactiae*, *H. influenzae*, and gram-negatives such as *Escherichia coli* and *Klebsiella pneumoniae* are responsible for most of the cases [1].

With increasing use of vaccines, accompanied by the possibility of serotype replacement [11, 12], the incidence and aetiology of community-acquired bacterial meningitis (CABM) are likely to change. An aging population and a growing use of immunosuppressive therapies may increase the susceptibility for bacterial meningitis. Therefore, further studies are needed to evaluate these changes in incidence and aetiology. Previous studies have generally been based on either International Classification of Disease (ICD) codes or only culture-proven cases of meningitis, but not both [6, 13, 14]. Furthermore, the emergence of Polymerase Chain Reaction (PCR) assays for detection of microbes, as a complement to cultures, has increased the possibility to identify causative pathogens, even if antibiotic therapy has been initiated prior to lumbar puncture. It is reasonable to suspect that previous studies may have underestimated the incidence of bacterial meningitis, and the actual proportions of causative pathogens may be different.

The aim of this study was to describe the incidence, temporal trend, aetiology and diagnostic modalities of CABM in a population-based study. A secondary aim was to investigate the serotypes of *S. pneumoniae* and *N. meningitidis* causing CABM, in relation to available vaccines.

## Methods

### Study design and setting

This study was a retrospective population-based cohort study in Skåne, southern Sweden, 2013–2023. Skåne has a population of approximately 1.4 million (2023). As Skåne is a peninsula, inhabitants are presumed to seek medical care in one of ten hospitals in the county, of which all but one share the same medical records system. Furthermore, all microbiological testing in Skåne is centralized and performed at the Department of Clinical Microbiology Skåne University Hospital, Lund.

This study was approved by the Swedish ethical review authority (reference id 2023-01914-01). All aspects of the study have been conducted in line with the Declaration of Helsinki.

### Participants and definitions

Patients of all ages admitted to a hospital in Skåne with CABM 2013–2023 were included in the study. Patients were primarily identified through records from the Department of Clinical Microbiology at Skåne University Hospital, Lund, which provided data of all cerebrospinal fluid (CSF) analyses with a positive culture, PCR, or antigen test during the specified time period. In addition, patients were identified through ICD-10 codes for bacterial meningitis, which were retrieved from the regional healthcare database. Exclusion criteria were either non-bacterial meningitis, mycobacterial infection, neuroborreliosis, syphilitic meningitis, healthcare-associated meningitis (defined as onset of symptoms > 48 h after admission), neurosurgery in the preceding 90 days, presence of a neurosurgical device (e.g. ventriculo-peritoneal shunt), primary admittance to a hospital outside of Skåne, or unavailable medical records. An episode of bacterial meningitis was defined as signs and symptoms consistent with bacterial meningitis (e.g. fever, headache, neck stiffness, altered mental status, and CSF pleocytosis) and either: (1) a positive CSF culture, CSF PCR, or CSF antigen test, or (2) an ICD-10 code consistent with bacterial meningitis. In case of difficulties regarding the inclusion of a specific patient, discussion was conducted between the authors.

### Patient data

Medical records were manually reviewed, according to a prespecified protocol, to extract patient data, including comorbidities, presenting symptoms, microbiological findings, and CSF-analyses. Serotype data was obtained from the Regional Office of Communicable Disease Control and Prevention.

### Aetiology

A positive CSF culture, CSF PCR or CSF antigen test were considered sufficient to determine aetiology. If a pathogen known to cause meningitis was identified in blood cultures, cultures or PCR in close connection with the meninges (e.g. ear or mastoid), or urine antigen test for *S. pneumoniae*, it was also deemed sufficient to determine aetiology, provided no conflicting microbiological findings were present. Cultures and PCR results considered to be contamination, which was based on the assessment by the attending physician in each case, were disregarded in the analysis. If there were no reasonable microbiological findings, such as culture-negative CABM based on clinical parameters and ICD-10 codes, the episode was considered of unknown aetiology.

### Microbiological testing

For CSF, the standard culture method used clinically during the study period included plating on agar plates in both aerobic and anaerobic atmosphere, as previously described [15]. During the study period, matrix-assisted laser desorption/ionization time-of-flight mass spectrometry (MALDI-TOF MS: Bruker Daltonics, using the Bruker MBT Compass library version most recent at the time of sample analysis) was used as the main method for species identification of cultured bacteria. In cases where species identification was not possible using MALDI-TOF MS, 16 S sequencing using Sanger methodology was used (an in-house method described in a previous publication [15]). Directed in-house PCR (alone or combined with 16 S amplification and sequencing) was used as the primary method, in conjunction with culture, for the identification of *N. meningitidis*, *H. influenzae*, and *S. pneumoniae* until March 2020, when the FilmArray Meningitis/Encephalitis panel (Bio-Fire Diagnostics, Biomérieux) was introduced. After the introduction of the FilmArray Meningitis/Encephalitis panel, the in-house PCR methods were used as complementary methods. CSF and urine antigen tests for *S. pneumoniae* were in use during the study period (both tests from Immuview, SSI Diagnostica and BinaxNOW, Abbot).

### Statistical analysis

Calculation of annual incidence rates was based on population size reported by Statistics Sweden (https://www.scb.se/en/). To increase comparability between settings, and to control for demographic changes of the population over time, age-standardised incidence rates based on the European standard population 2013 [16] were also calculated. For incidence rates in different age groups, only crude incidence rates were calculated. The chi-square test was used to compare differences in categorical data. A 95% confidence interval (CI) was used and a p-value of < 0.05 was considered statistically significant. Statistical analyses were performed using R statistical software version 4 (R Foundation for Statistical Computing (www.r-project.org)).

## Results

### Episodes and patient characteristics

**Fig. 1:**
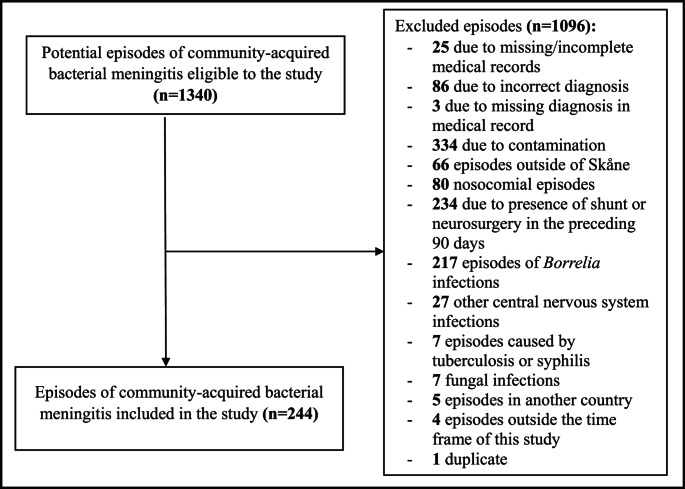
Flowchart of included episodes

A total of 1340 episodes were assessed for potential inclusion. Ultimately, 244 episodes of CABM in 238 individuals were included in the study (Fig. 1). Four individuals had two episodes of CABM during the study period, and one had three episodes. Minimal, median, and maximal time to the recurrences of CABM were 10, 102, and 298 weeks, respectively. Out of the 244 CABM episodes, 22.5% occurred in children and 77.5% in adults, and the median age was 60 years (interquartile range 22–71 years). 54.5% of all episodes occurred in males (Table 1).

**Table 1:** Patient characteristics

	*n*=244	%^a^
Background		
Sex, male	133	54.5
Age, median (IQR)	60 (22-71)	*na*
Newborns (<3 months)	29	11.9
Young children (3 months - 4 years)	14	5.7
Older children (5–17 years)	12	4.9
Adults (18–64 years)	89	36.5
Elderly (≥65 years)	100	41
Charlson comorbidity index, median (IQR)	2 (0-3)	*na*
Diabetes	25	10.2
Previous CVI or TIA	15	6.1
Previous myocardial infarction	13	5.3
Heart failure	9	3.7
Chronic obstructive pulmonary disease	8	3.3
Risk factors for CABM		
Transplanted^b^	2	0.8
Immunosuppression^c^	16	6.6
Cochlear implant	0	0
Asplenia^d^	4	1.6
Previous bacterial meningitis	8	3.3
CSF leakage	10	4.1
Alcoholism	8	3.3
Anatomical risk^e^	20	8.2
Concomitant focus of infection		
Otitis^f^	43	17.6
Sinusitis	12	4.9
Pneumonia	18	7.4
Mastoiditis	1	0.4
Pharyngitis	5	2.0
Tooth infection	2	0.8
Endocarditis	2	0.8
Spondylodiscitis/epidural abscess	4	1.6
Septic arthritis	1	0.4
Otitis^f^ and mastoiditis	11	4.5
No concomitant focus found	145	59.4

CABM: community-acquired bacterial meningitis, CSF: cerebrospinal fluid, CVI: cerebrovascular insult, IQR: interquartile range, na: not applicable, TIA: transient ischemic attack

^a^ratios were calculated based on available data

^b^solid organ and stem cell transplantation

^c^treatment with medications from ATC-code L01 or L04, corticosteroid treatment equivalent to ≥10 mg of prednisolone daily, or primary immunodeficiency

^d^congenital or due to splenectomy

^e^congenital malformations in connection with meninges, previous skull fracture, or previous skull/brain surgery without surgical implants

^f^including acute otitis media and unspecified otitis

The most prevalent comorbidities were diabetes mellitus (10.2%), cerebrovascular disease (6.1%), and myocardial infarction (5.3%). Immunosuppression (immunosuppressive drugs or primary immunodeficiency) was present in 16 episodes (6.6%). In twenty (8.2%) episodes, there was a documented anatomical risk factor, such as previous skull fracture or congenital malformation, and 10 (4.1%) had a history of CSF leakage. Notably, four (80%) of the individuals with recurrent CABM had an anatomical risk factor at the time of their first episode, and two (40%) had a documented history of CSF leakage.

In most cases (59.4%), no concomitant focus of infection could be established other than meningitis. Otitis (with or without mastoiditis) was most common (22.1%), followed by pneumonia (7.4%) and sinusitis (4.9%).

### The Microbiological diagnosis of community-acquired bacterial meningitis

**Fig. 2:**
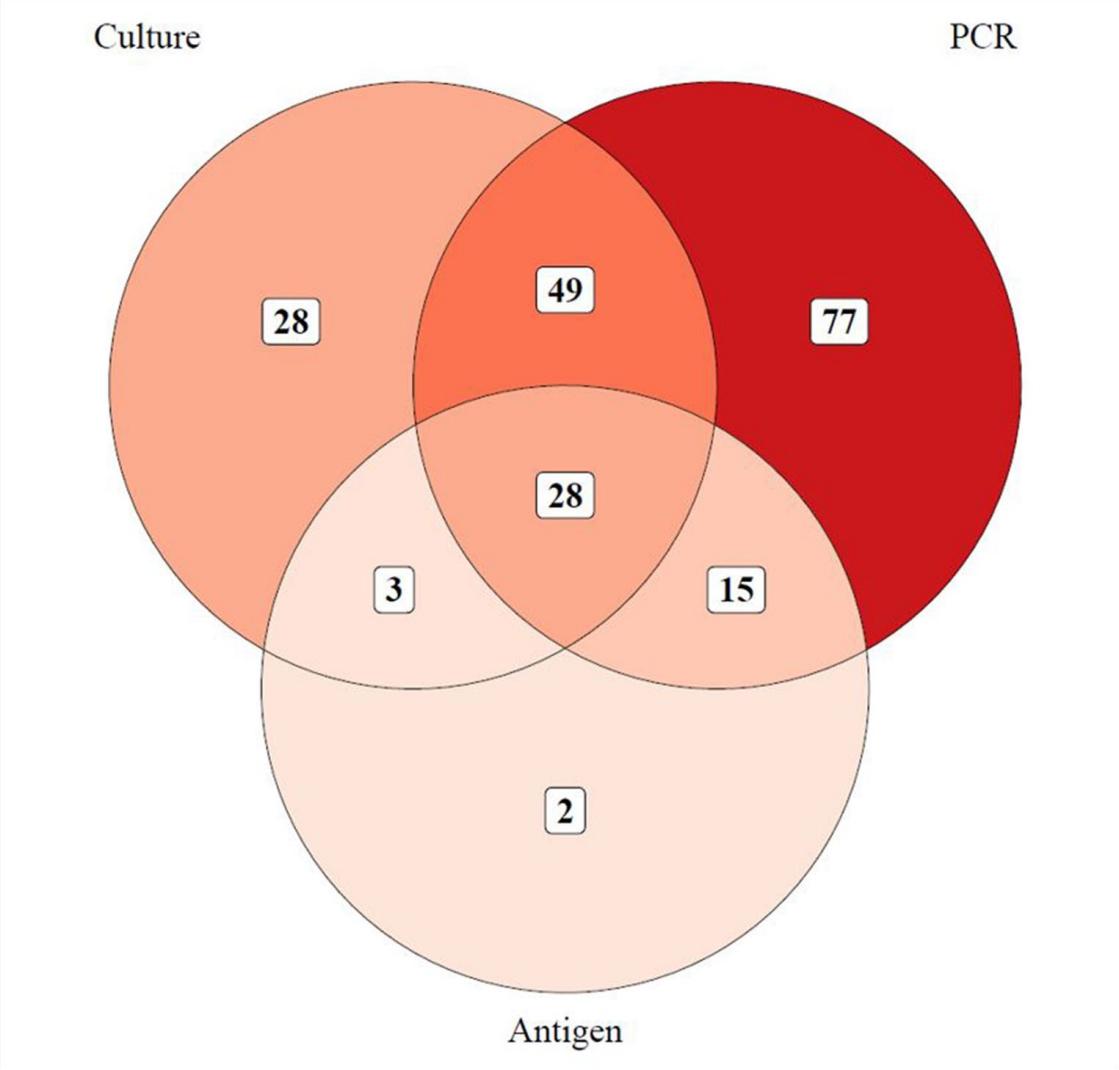
Venn diagram of microbiological diagnostic modalities in cerebrospinal fluid PCR: Polymerase chain reaction. Distribution and overlap of microbiological cerebrospinal fluid tests in episodes with at least one positive test (*n*=202). Antigen tests were only directed against *Streptococcus pneumoniae*. Forty-two episodes without microbiological findings in cerebrospinal fluid are not included in the diagram

Lumbar puncture was performed in 233 (95.5%) episodes, CSF cultures were obtained in 230 episodes, and CSF PCR were obtained in 217 episodes. CSF cultures were positive in 47% (108/230) of episodes. The proportion of positive CSF cultures was significantly higher when lumbar puncture was performed prior to administration of antibiotics, 60.3% (41/68) vs. 41.4% (67/162), OR = 2.15 (95% CI 1.21–3.84, *p* = 0.009). CSF PCR was positive in 77.9% (169/217) of episodes. When CSF PCR was positive, there was 100% concordance between CSF cultures, CSF PCR and blood cultures, when disregarding contaminants. In 28.7% (70/244) of all episodes, a positive CSF PCR and blood culture were obtained, when CSF cultures were negative or absent. Among the 101 episodes with a positive CSF culture and/or CSF PCR for S. pneumoniae, CSF antigen tests were performed in 47 episodes, of which 44 (93.6%) were positive. In 44.3% (108/244) of all episodes, the diagnosis was based on positive CSF cultures (regardless of CSF PCR and antigen test), 37.7% (92/244) was based on positive CSF PCR (with negative CSF cultures), 0.8% (2/244) on CSF antigen test (with negative CSF culture and PCR), and 17.2% (42/244) on clinical parameters (negative CSF culture, PCR, and antigen test) (Fig. 2). Characteristics of the episodes diagnosed by clinical parameters are presented in Supplementary table S1.

### Aetiology of community-acquired bacterial meningitis

A causative pathogen was identified in 227 (93%) episodes (Table 2). Two hundred and two (82.8%) were identified by CSF culture, CSF PCR, or CSF antigen test, 21 (8.6%) by positive blood cultures, one (0.4%) by culture from subdural pus, one (0.4%) by urine antigen test, one (0.4%) by ear culture (through paracentesis), and one (0.4%) by PCR from maxillary sinus flush. The aetiology was unknown in 17 episodes (7%). Of these, 14 had negative CSF-samples and blood cultures, two did not undergo lumbar puncture and had negative blood cultures, and one was confirmed by autopsy. For the entire study population (*n* = 244), the most common pathogens were *S. pneumoniae* (*n* = 114, 46.7%), *S. agalactiae* (*n* = 24, 9.8%) and *N. meningitidis* (*n* = 23, 9.4%). For newborns (age < 3 months), *S. agalactiae* was most prevalent, accounting for 20 (69%) episodes, followed by *S. pneumoniae* and *E. coli*, each accounting for 3 (10.3%) episodes. *S. pneumoniae* was the most common pathogen in all other age groups, accounting for 50-58.3% of episodes. Listeria meningitis only occurred in adults, and 80% of these episodes occurred in individuals ≥ 65 years.

**Table 2:** Aetiology of community-acquired bacterial meningitis in Southern Sweden 2013–2023

	All ages		Newborns (< 3 months)		Young children (3 months–4 years)		Older children (5–17 years)		Adults (18–64 years)		Elderly (≥ 65 years)	
	*n* = 244	%	*n* = 29	%	*n* = 14	%	*n* = 12	%	*n* = 89	%	*n* = 100	%
Aetiology found	227	93	29	100	14	100	12	100	80	89.9	92	92
*Streptococcus pneumoniae*	114	46.7	3	10.3	7	50	7	58.3	46	51.7	51	51
*Neisseria meningitidis*	23	9.4	2	6.9	4	28.6	4	33.3	10	11.2	3	3
*Listeria monocytogenes*	15	6.1	0	0	0	0	0	0	3	3.4	12	12
*Haemophilus influenzae*	15	6.1	0	0	2	14.3	0	0	6	6.7	7	7
*Staphylococcus aureus*	7	2.9	0	0	0	0	0	0	3	3.4	4	4
*Escherichia coli*	6	2.5	3	10.3	0	0	0	0	0	0	3	3
*Streptococcus agalactiae*	24	9.8	20	69	0	0	0	0	1	1.1	3	3
Other streptococci	14	5.7	0	0	0	0	1	8.3	6	6.7	7	7
Other bacteria	9	3.7	1	3.4	1	7.1	0	0	5	5.6	2	2
Unknown aetiology	17	7	0	0	0	0	0	0	9	10.1	8	8

### Incidence rates and trend of community-acquired bacterial meningitis during the study period

**Fig. 3:**
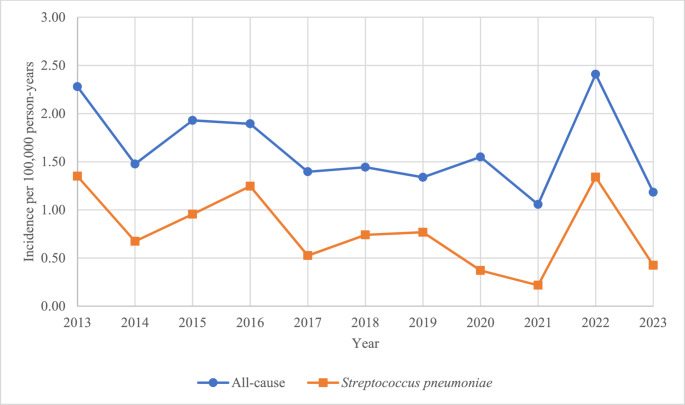
Age-standardised incidence rates for community-acquired bacterial meningitis in southern Sweden 2013–2023

The annual age-standardised incidence rate for all-cause CABM ranged from 1.06 (year 2021) to 2.41 (year 2022) per 100,000 person-years (Fig. 3). The mean age-standardised incidence rate throughout the study period was 1.63 per 100,000 person-years. There was a trend towards declining incidence during the study period. In general, the crude and age-standardised incidence rates were similar. The mean crude incidence rates for children, all adults, and elderly only (≥ 65 years) were 1.74, 1.62, and 3.5 per 100,000 person-years, respectively. For *S. pneumoniae*, age-standardised incidence rates ranged from 0.22 (year 2021) to 1.35 (year 2013) per 100,000 person-years. A trend towards declining incidence of pneumococcal meningitis was seen, similar to all-cause CABM (Fig. 3). For pathogens other than *S. pneumoniae*, mean age-standardised incidence rates ranged from 0.04 to 0.15 per 100,000 person-years (Supplementary table S2). Examining temporal occurrences of meningococcal meningitis, no definitive signs of clustered episodes were evident during the study period. Two episodes were confirmed within six days from each other; however, serotype data were not available in one of the episodes.

### Serotypes of Streptococcus pneumoniae and neisseria meningitidis

Serotype data was available for 107/114 episodes (93.7%) of pneumococcal meningitis (Table 3). Thirty-one different pneumococcal serotypes were identified, of which serotype 8, 3 and 22 F were the most common, accounting for 12.1%, 11.2% and 8.4% of the episodes, respectively. In one episode, two different serotypes (3 and 22 F) were identified in the same blood culture.

**Table 3:** Distribution of pneumococcal serotypes causing community-acquired bacterial meningitis in southern Sweden 2013

Serotypes included in available vaccines^a^	*n*	%	Serotypes not included in available vaccines^a^	*n*	%
3	12	11.2	6 C	4	3.7
4	2	1.9	7	1	0.9
6 A	1	0.9	7 C	1	0.9
6B	1	0.9	10 F	1	0.9
7 F	1	0.9	15 A	7	6.5
8	13	12.1	15 C	4	3.7
9 N	2	1.9	16 F	1	0.9
9 V	1	0.9	18B	1	0.9
10 A	3	2.8	23 A	8	7.5
11 A	5	4.7	23B	4	3.7
15B	5	4.7	24 F	2	1.9
17 F	1	0.9	35B	1	0.9
19 A	7	6.5	35 F	3	2.8
20	1	0.9	38	1	0.9
22 F	9	8.4	Total	39	36.4
23 F	1	0.9			
33 F	2	1.9			
22 F and 3	1	0.9			
Total	68	63.6			

^a^ the 23-valent pneumococcal polysaccharide vaccine and the 20-valent pneumococcal conjugate vaccine combined.

For meningococcal meningitis, serogroup data was available for 21/23 episodes (91.3%). Serogroup B was most prevalent, accounting for 12 episodes (57.1%). Serotype C, Y, and W135 each accounted for 3 episodes (14.3% each).

## Discussion

To the best of our knowledge, this is the first study of community-acquired bacterial meningitis to combine case findings from ICD-codes and microbiological tests. The aim was to present updated epidemiological data, as well as the contribution of different diagnostic modalities, on community-acquired bacterial meningitis in our setting. The mean age standardised incidence rate during the study period was 1.63 per 100,000 person-years, and there was a trend towards declining incidence during the study period. *S. pneumoniae* was the most common pathogen.

### The Microbiological diagnosis of community-acquired bacterial meningitis

The absence of definite diagnostic criteria for bacterial meningitis presents difficulties when comparing mode of diagnosis and the diagnostic yield of different tests. In the absence of positive CSF cultures, characteristic CSF findings such as elevated CSF leukocytes with polymorphonuclear dominance has sometimes been applied as inclusion criteria in previous studies [8]. However, in some cases of bacterial meningitis, there is a lymphocytic dominance in CSF, or even normal leukocyte counts [17–19]. Furthermore, sometimes lumbar puncture is delayed or cannot be performed. Therefore, using too strict inclusion criteria based on positive CSF culture and/or other specific CSF findings, may lead to the exclusion of a significant proportion of cases in studies of bacterial meningitis. In our study, we used wider inclusion criteria with the aim to represent the true spectrum of community-acquired bacterial meningitis. This may account for a lower proportion of CSF culture-positive cases, compared to previous studies [20]. Another explanation may be due to the large proportion of episodes where lumbar puncture was performed after administration of antibiotics, which has been shown to decrease the number of positive CSF cultures [17]. On the other hand, a majority of the episodes in our study had a positive CSF PCR, with excellent concordance with CSF and blood cultures. CSF PCR could therefore improve the diagnostic yield of lumbar puncture, especially in combination with blood cultures, in situations where the procedure is delayed.

Notably, 334 potential episodes were excluded due to contamination of CSF samples (cultures and PCR). This may reflect that the samples often are retrieved in stressful situations where sterility is not maintained. It highlights the need to interpret microbiological CSF findings with caution, and always in relation to other laboratory tests and the clinical situation.

### Aetiology

The aetiology of CABM in our study was mainly, but not exclusively, based on CSF-related microbiological test. *S. pneumoniae* was the most common aetiology (46.7%) found, in accordance with previous studies where the percentage ranged from 44.9 to 72% [6, 8, 13, 14]. The second most prevalent aetiology, *S. agalactiae*, is primarily driven by bacterial meningitis episodes in newborns. In a study by Polkowska et al. [6], including only CSF culture-positive cases of five common aetiologies, *S. agalactiae* accounted for 10.3% of the episodes, compared to 9.8% in our study. In a study of neonatal (age < 3 months) meningitis in the UK [21], *S. agalactiae* caused 50% of cases, somewhat less frequent than in our study (69%). Overall, our distribution of aetiology is very similar to the numbers reported for CABM 2009–2014 in a previous Swedish study [14].

### Incidence

The all-cause and pneumococcal incidence rates in our study showed concordance with an overall decreasing trend during the study period. Several recent studies of bacterial meningitis also report a declining incidence [8, 14, 22]. Given that pneumococcal meningitis constituted nearly half of all episodes, it likely serves as the primary driver of all-cause CABM trends observed in our clinical setting. A previous study [23] in Skåne reported a decreasing incidence of bloodstream pneumococcal infections 2006–2019, in line with our findings.

Notably, there was a peak in incidence for both all-cause and pneumococcal CABM in year 2022 in our study. One possible explanation is that Sweden lifted many COVID-19 pandemic restrictions in early 2022, likely leading to increased social interactions. Another explanation might be the result of covariance with influenza virus, as previously described [24], since the incidence rates for influenza virus during winter season in Sweden 2021–2022 and especially 2022–2023 were unusually high [25]. The incidence rates for pathogens other than *S. pneumoniae* in our study are insufficient for definitive trend analysis.

Direct comparisons of incidence rates are somewhat challenging, due to differences in study design and settings. Studies based on specific microbiological criteria will likely be limited by missing cases, especially those where an aetiology cannot be identified. On the other hand, studies based on ICD-codes are influenced by how bacterial meningitis is diagnosed in clinical practice in that setting, and the diagnosis may be made with insufficient evidence. In prospective studies, inclusion may be limited by declined consent from patients and a lack of reporting from clinicians. Moreover, incidence rates are sometimes reported as a mean for the study period, and sometimes for individual years, further complicating comparisons. All things considered, recent heterogeneous studies of CABM and unspecified bacterial meningitis report incidence rates ranging from 0.7 to 2.7 per 100,000 person-years [6–8, 14, 22, 26], which seems to be in line with our findings.

### Serotypes

In Sweden, pneumococcal vaccination has been a part of the national childhood vaccination programme since 2009. The 10-valent pneumococcal conjugate vaccine (PCV10) has mainly been used, although sometimes interspersed with PCV13. Vaccine coverage in children have been high, > 93% since 2016 [27]. Furthermore, pneumococcal vaccinations (generally the 23-valent pneumococcal polysaccharide vaccine (PPSV23)) have been recommended to risk groups including elderly (age ≥ 65 years) since 1994 [28]. Unfortunately, vaccination status in our study population is unknown, making it difficult to assess the causality between vaccination and serotype distribution in our study. Moreover, some serotypes are prone to cause breakthrough infections despite vaccination, such as serotype 3 [29]. However, in our study, only six (5.6%) episodes of pneumococcal meningitis were caused by serotypes included in PCV10, with five of these six cases occurring in adults. Before pneumococcal vaccines were introduced, serotypes included in PCV10 accounted for > 50% of invasive pneumococcal disease including meningitis [30, 31]. This indicates a vaccine effect in our study population.

Thirty-nine (36.4%) episodes were caused by serotypes for which there are no available vaccines today. This may imply the need for further vaccine development, in addition to public health measurements to increase vaccine availability and adherence in the population.

Vaccination against *N. meningitidis* is not included in the current vaccination programme, but is recommended to certain risk groups [32]. All meningococcal serogroups identified in this study could potentially be prevented by available vaccines. However, the low incidence rate may not motivate universal vaccination, as the cost of general vaccination must be weighed against the incidence and impact of meningococcal meningitis. This complex public health assessment ultimately falls within the purview of health policy authorities, who must weigh these factors alongside other public health priorities.

### Strengths and limitations

The strength of this study is the comprehensive case finding from two different sources, in combination with a population-based design in a suitable setting, ensuring a minimal number of missing episodes. Moreover, compared to previous studies, we also included episodes of CABM based on CSF PCR, CSF antigen and clinical parameters, to cover the full spectrum of CABM. However, this study also presents some limitations. First, the retrospective design presumably results in some missing data and lower yield in microbiological findings, since CSF cultures, PCR, and antigen test were not performed in all patients. Second, 25 episodes potentially eligible for the study were excluded due to missing or incomplete medical records, which is approximately 10% of our study population. Third, our slightly wider criteria for inclusion and determination of aetiology than previous studies [6, 8, 13], might overestimate the incidence rates and actual number of cases per pathogen.

### Further implications

To increase the diagnostic yield of CSF cultures in clinical practice, lumbar puncture should preferably be performed prior to antimicrobial treatment. Also, the use of CSF PCR should be emphasized to improve diagnostic accuracy, especially in cases where antibiotic treatment precedes lumbar puncture. Considering incidence and aetiology, the generalisability of our results is reasonably limited to countries with similar age distribution and vaccination patterns. Nevertheless, our data shows that over a third of pneumococcal meningitis is caused by serotypes for which there are no available vaccines today, indicating the need for development of new vaccines. Future studies are needed to continuously monitor the dynamics of shifting aetiologies, serotypes, incidence rates, and antibiotic susceptibility of community-acquired bacterial meningitis.

## Conclusions

Pneumococcal meningitis is the primary driver of incidence of all-cause CABM in southern Sweden, and the trend during the study period indicate decreasing incidence. Most cases of pneumococcal meningitis are caused by serotypes for which there are available vaccines. CSF PCR in combination with blood cultures are useful to determine aetiology and guide antibiotic therapy in the absence of positive CSF cultures.

## Supplementary Information

Below is the link to the electronic supplementary material.

Supplementary Material 1

## Data Availability

The datasets generated and analysed during the current study are not publicly available due to confidentiality laws and possible compromise of individual privacy.
