# Supplementary material for: Community-acquired bacterial meningitis in Southern Sweden 2013–2023: a population-based study of incidence, aetiology and diagnostic yield
Source: Eur J Clin Microbiol Infect Dis. 2025 Oct 1;45(1):103–12. doi: 10.1007/s10096-025-05247-w (PMC12872648; doi:10.1007/s10096-025-05247-w)
Supplement: Supplementary file 1 — Supplementary Material 1 [file 10096_2025_5247_MOESM1_ESM.docx]

**Supplementary material**

*Journal*

European Journal of Clinical Microbiology & Infectious Diseases

*Title*

Community-acquired bacterial meningitis in southern Sweden 2013–2023: a population-based study of incidence, aetiology and diagnostic yield

*Authors*

Tobias West^1,2^, Robin Carlander^1^, Torgny Sunnerhagen^1,3^, Gustav Torisson^4,5^, Oskar Ljungquist^1,6^

*Affiliations*

1. Division of Infection Medicine, Department of Clinical Sciences, Faculty of Medicine, Lund University, Lund, Sweden

2. Department of Internal Medicine, Helsingborg hospital, Helsingborg, Sweden.

3. Department of Clinical Microbiology, Infection Control and Prevention, Skåne University hospital, Lund, Sweden.

4. Clinical Infection Medicine, Department of Translational Medicine, Lund University, Malmö, Sweden.

5. Department of Infectious Diseases, Skåne University hospital, Malmö, Sweden.

6. Department of Infectious Diseases, Helsingborg hospital, Helsingborg, Sweden.

*Corresponding author*

Tobias West (tobias.west@med.lu.se)

**Supplementary table S1. Characteristics of episodes with diagnosis based on clinical parameters**

Summary of the basis for diagnosis for episodes without microbiological findings in CSF. All episodes had symptoms consistent with bacterial meningitis, such as altered mental status, headache, neck stiffness, fever, and seizures.

**CSF:** cerebrospinal fluid

^a^ a CSF glucose level <1.9 mmol/L, a CSF-blood glucose ratio <0.23, a CSF protein level >2.2 g/L, >2000 x 10^6^/L CSF leukocytes, or >1180 x 10^6^/L CSF polymorphonuclear leukocytes. ^b^ four episodes had blood cultures positive for bacteria known to cause meningitis.

|  | ***n*=42** | **%** |
| --- | --- | --- |
| Diagnosis verified by autopsy | 1 | 2.4 |
| Meets ≥1 Spanos criteria^a^ for bacterial meningitis | 14 | 33.3 |
| Elevated leukocytes with polymorphonuclear dominance in CSF^b^ | 13 (4) | 31 |
| Elevated leukocytes with lymphocytic dominance in CSF, with multiple bacteria cultured from subdural pus | 1 | 2.4 |
| Elevated leukocytes with lymphocytic dominance in CSF, with *Listeria monocytogenes* in blood cultures | 3 | 7.1 |
| Lumbar puncture not performed; blood cultures positive for bacteria commonly causing meningitis | 8 | 19 |
| Lumbar puncture not performed; blood cultures negative | 2 | 4.8 |

**Supplementary table S2. Mean age-standardised incidence rates for community-acquired bacterial meningitis in southern Sweden 2013–2023**

| **Pathogen** | **Incidence per 100,000 person-years** |
| --- | --- |
| *Streptococcus pneumoniae* | 0.78 |
| *Streptococcus agalactiae* | 0.14 |
| *Neisseria meningitidis* | 0.15 |
| *Haemophilus influenzae* | 0.10 |
| *Listeria monocytogenes* | 0.10 |
| Other streptococci^a^ | 0.10 |
| Other bacteria^b^ | 0.06 |
| *Staphylococcus aureus* | 0.05 |
| *Escherichia coli* | 0.04 |
| Unknown aetiology | 0.12 |

^a^ including *S. intermedius, S. pyogenes*, *S. dysgalactiae*, *S. equi*, *S. anginosus*, *S. mitis*, and *S. salivarius*.

^b^ including *Klebsiella pneumoniae*, *Pseudomonas aeruginosa*, *Enterococcus faecalis*, *Capnocytophaga canimorsus*, *Salmonella typhimurium*, *Fusobacterium necrophorum*, and mixed anaerobic bacteria

|  |
| --- |
|  |
|  |
|  |
|  |
|  |
|  |
|  |
|  |
